# Promoting Effective Self-Management of the Gluten-Free Diet: Children’s and Adolescents’ Self-Generated Do’s and Don’ts

**DOI:** 10.3390/ijerph192114051

**Published:** 2022-10-28

**Authors:** Sonya Meyer

**Affiliations:** Department of Occupational Therapy, Ariel University, Ariel 4077603, Israel; sonyam@ariel.ac.il; Tel.: +972-3-645-3123

**Keywords:** celiac, cognition, food-related activity, self-management

## Abstract

Celiac disease (CD) is a chronic health condition treated by managing a lifelong, strict, and demanding gluten-free diet. Managing the diet entails effective use of self-management skills. This study aimed to explore self-generated procedures children and adolescents with CD in Israel perform when participating in food-related activities considering their self-management skills and health requirements. Participants included children and adolescents with CD, aged 8 to 18 years, that had been diagnosed more than 6 months prior to the study. Parents completed a demographic questionnaire and reported their child’s constancy in adherence to the diet. Children and adolescents were asked to share the things they do themselves to prepare for participating in the various activities. Responses were qualitatively analyzed, and common themes were identified and categorized using directed analysis. Participants were 126 children and adolescents (*M_age_* = 12.33 yr, *SD* = 2.85), 67.5% of whom had been diagnosed more than 3 yr prior to the study. Based on parents’ reports, almost all (97.6%) participants “always adhered” to the diet. A total of 10 categories were defined from the qualitative responses describing 125 do and don’t actions used by the children and adolescents to self-manage their diet. The do and don’t actions encompass cognitive planning far beyond the mere act of avoiding gluten. These actions can serve as an initial database of suggested strategies to support acquiring independent self-management. Understanding the cognitive complexity of routinely carrying out the diet while actively participating in everyday activities can assist health professionals in building support and intervention programs, promoting effective self-management, and facilitating optimal adherence to the diet.

## 1. Introduction

Celiac disease (CD) is a chronic immune-mediated condition characterized by inflammatory damage to the small intestine. It is an increasingly common global health condition with a worldwide prevalence of around 1% [1,2,3]. Managing a lifelong, strict, gluten-free diet (GFD) is currently the only available effective and accepted treatment for CD [2,4]. Adherence to a GFD is demanding, and the adherence rate can be as low as 45% and among children and adolescents, from 23% to 98%, with incomplete adherence more common in adolescents [5,6]. Thus, the importance of understanding the factors associated with supporting adherence and maintaining the diet is emphasized [7].

As children grow into adolescence and advance into young adulthood, the disease management shifts from pediatric to adult care [8]. Apart from the transition from the health care system and providers’ perspectives, adolescence represents a complex evolving period of transition from childhood to adulthood characterized by unique health and developmental issues [9]. From a developmental perspective, this transitional stage leads adolescents to expand their active day-to-day circles to environments further from their home and especially their social environments [10]. Such activities include, for example, social gatherings, outings, parties, camps, and eating out [11,12,13]. As such, as children with CD transition into adolescents with CD, they gradually must take over the decision-making responsibility from their parents and independently self-manage their health condition [13].

Self-management is the interaction of health behaviors and related processes and the lifelong tasks of actively caring for oneself and living well with a chronic condition [14,15]. In the CD context, the required health behavior is predominantly managing a GFD. Attaining these tasks involves cognitive skills and transition planning, including developing independence in self-management tasks [13,16]. Children and adolescents diagnosed with CD need to maintain a GFD in often-complex food-related daily life situations. Complexity is especially apparent when children and adolescents are away from the comfort and familiarity of their home environments [16].

To fully participate like their friends in a range of daily activities while managing their health requirements, children and adolescents with CD must find solutions, implement cognitive strategies, and develop new habits and routines in varied environments [16,17]. Fishman et al. created an innovative 18-item list of patient-centered CD skill benchmarks to promote self-management skills [13]. Health professionals, parents of children with CD, and adults with CD suggested the benchmarks, highlighting the significance of transition planning to create educational tools and guidance as undoubtedly essential. However, education alone is insufficient to promote the acquisition of self-management skills, knowledge, and confidence for self-management. Instead, self-management is gained through conscious and planned engagement in specifically structured real-life contexts [18,19].

Self-reports are considered an important source of information to measure nutrition and health behaviors [20]. Evidence shows that information and strategies are better learned when self-generated than when provided by others [21]. Additionally, self-management interventions must be self-tailored, focusing on how the individual perceives disease-related issues to support managing the condition [14,22]. Thus, analyzing activities through client-specific subjective perspectives, based on how clients engage in activities in their own contexts, is vital to understanding the activities’ demands [23]. Therefore, obtaining subjective perspectives from 102 children and adolescents with CD could contribute to filling the gap between parental and professional perspectives and self-reported perspectives. Moreover, further the understanding of how they plan and carry out their thinking processes and their actions toward participating in complex, everyday food-related activities is vital [12]. The purpose of the current study was to:

(1) Explore self-generated procedures children and adolescents with CD perform when participating in food-related activities in light of their self-management skills and health requirements;
(2) Present an initial database of suggested strategies supporting self-management among children and adolescents with CD using self-management do’s and don’ts that they generated;
(3) Compare the strategies used by children versus adolescents and by time passed since diagnosis.

## 2. Materials and Methods

### 2.1. Participants and Procedure

Participants were 126 children and adolescents (82 girls, 44 boys) diagnosed with CD. They volunteered to participate in the study by responding to advertisements via local celiac associations, local online support groups, and social media from February to September 2015. The inclusion criteria were children and adolescents aged 8 to 18 yr with CD diagnoses confirmed by a physician no less than 6 months prior to the study and Hebrew language proficiency. Children diagnosed with physical or neurological disabilities were excluded. The Ethics Committee on Research with Human Participants, University of Haifa, Faculty of Social Welfare & Health Sciences approved the study (026/15, 18 January 2015). All children and adolescents and their parents provided written informed consent.

Parents completed an online demographic questionnaire, as part of a larger study that included several parent and self-report questionnaires, including, inter alia, a previously published questionnaire, the Celiac Disease-Children’s Activities Report (CD-Chart) (i.e., meals at home, family meals/events, meals on family vacations, eating out with friends, food served at a friend, meals at an overnight camp/trip, eating treats handed out by the teacher, special food activities at school, and meals during overnight school field trips) [12]. For the current study, after completing the CD-Chart, the children and adolescents were asked to recount the things, methods, techniques, or procedures they do to prepare for participating in the various food-related activities: “Thinking about the various food-related activities in the questionnaire, which of the things you mentioned do you do yourself to prepare for participating in the various activities?” The children and adolescents were encouraged to express up to five actions they ensured they did or not do. Their responses were audio-taped and transcribed.

Level of adherence to a GFD was obtained via a parent report as part of the demographic questionnaire, rated from 1 = always adhere to 5 = never adhere to the diet. In addition, children and adolescents completed the Birmingham Celiac Disease Self-Management Questionnaire (CD-SMQ) [24], a four-item questionnaire for assessing self-reported gluten intake in home and out-of-home settings. The frequency of gluten intake or adhering to a GFD is rated on a 5-point Likert scale (from 1, never to 5, all the time). For use in this study, translation and back-translation were performed, and internal reliability for the translated version was found to be acceptable (Cronbach’s coefficient α = 0.75).

### 2.2. Data Analysis

**Figure 1:**
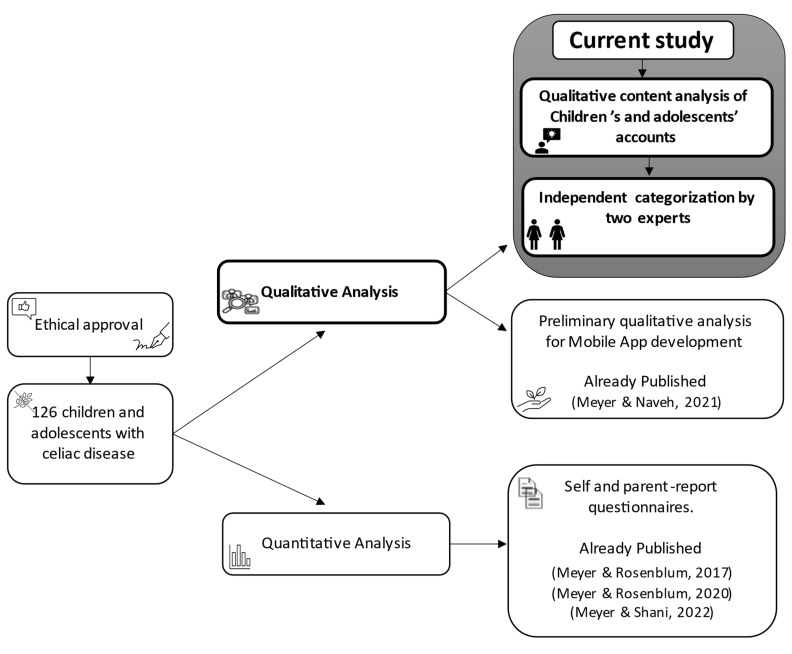
Methodology flowchart.

Quantitative demographic data were analyzed with IBM SPSS Statistics software (ver. 26) to calculate means and standard deviations (*SDs*) and describe the sample demographic characteristics. The qualitative data were initially analyzed in the original language, resulting in a list of 464 actions. Fluent in both languages, the author translated the results to English to ensure accuracy and maintain the nature of the content. After excluding duplicate responses, 125 actions were included for analysis. Similar responses that expressed different subtle nuances were included. The data were analyzed, and common themes were identified and categorized via directed analysis [25]. Partial results of this data were previously published, after initial analysis, accompanied by some examples only [26]. The content was read repeatedly until a categorization pattern was identified. Then, the content was sorted into categories. A second expert clinician and researcher in the child and adolescent development field also independently sorted the content into the 10 identified categories. Some actions could be associated with more than one action category; thus, the most suitable category was chosen according to the context of the action. The analysis process was followed by consultation concerning minor differences, and a consensus was obtained through mutual discussion to obtain expert agreement. The procedure is illustrated in Figure 1.

To compare answers between children and adolescents, participants were divided into two age groups, children aged 8 to 11 yr (*n* = 61) and adolescents aged 12 to 18 yr (*n* = 65), based on the acceptable definition of adolescence in Israel [27]. Pearson’s chi-square test was used to compare the action category use by age group and by time passed since diagnosis.

## 3. Results

Participants included 82 girls and 44 boys, aged 8 to 18 years (*M* = 12.33 years, *SD* = 2.85), diagnosed with CD by biopsy (89.7%) or blood tests (10.3%). The majority (88.9%) had been diagnosed before the age of 11 yr; 67.5% had been diagnosed more than 3 yr prior to the study. Others were diagnosed 1 to 3 yr (29.4%) and 6 months to 1 yr prior to the study (3.2%). Based on the parental report, almost all (97.6%) participants “always adhered” to a GFD, and the remaining 2.4% (three participants) “often adhered” to the diet. According to the CD-SMQ, the adolescents reported sticking to the diet all of the time during in- (95.2%) and out- (89.7%) of-the-home situations, and never knowingly eating food containing gluten at home 90.5% of the time and 83.3% of the time when away from home.

Participants were students in 2nd to 12th grade in mainstream schools, with 54.0% in elementary school (grades 1–6) 24.6% in middle school (grades 7–9), and 21.4% in high school (grades 10–12). Most participants were city residents (69%), and the remaining participants resided in various community settings. The family income was mostly high (63.5%) or average (25.7%), as defined according to Israeli Central Bureau of Statistics classification [28].

**Figure 2:**
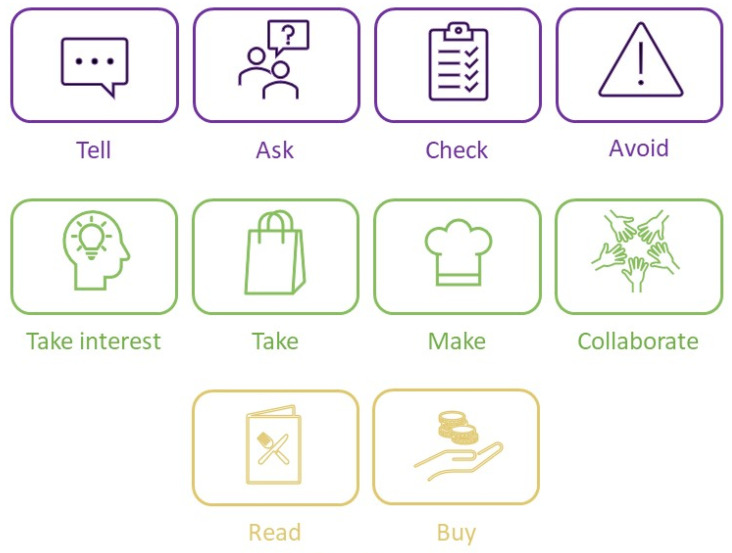
Managing celiac disease: do and don’t actions.

The qualitative accounts shared by the children and adolescents were categorized into 10 categories (Figure 2) describing their actions when preparing to participate in food-related activities.

Table 1 lists the 125 do and don’t actions the children and adolescents generated. The first category, *Tell*, and the second category, *Ask*, include 30 and 29 actions, respectively. *Tell* consists of actions in which the children and adolescents tell, inform, and convey information and requests to their parents, friends, service providers, authority figures, and others (e.g., “Tell others what I can and can’t eat”). *Ask* consists of questions directed to other people to ensure or inquire about their needs for safe participation while maintaining a GFD (e.g., “Ask the event organizer if there are any gluten-free accommodations”). The third category, *Check*, comprises 17 actions that promote finding and confirming information before deciding and choosing to participate safely in an activity while maintaining a GFD (e.g., “Check information online, on social media” or “Check and then double-check”). The fourth category, *Avoid*, encompasses 12 actions that remind or help the participant to avoid gluten when participating in food-related activities. The fifth category, *Take interest*, consists of eight actions that reflect an interest in celiac disease, dietary requirements, and responsibility while participating in food-related activities (e.g., “Keep up-to-date on celiac information”). The sixth category, *Take*, contains seven actions of bringing gluten-free food or substitutes to an activity (e.g., “Take gluten-free food in a cooler bag”). The seventh category, *Make*, refers to six cooking or baking actions or preparing other items (e.g., “Prepare reminder notes for myself”). The eighth category, *Collaborate*, incorporates six actions that involve doing an activity with someone else (e.g., “Take part in planning a social gathering”). The ninth category, *Read*, includes six actions related to reading information about celiac disease and gluten-free food labels (e.g., “Look for a ‘“gluten-free’” sign or icon”). Finally, the tenth category, *Buy*, contains four actions related to purchasing gluten-free products.

As presented in Table 2, this was followed by a comparison between the two age groups (children and adolescents), which showed significant differences in three action categories, revealing that the adolescents shared use of *ask* and *make* actions significantly more than the children, and the children reported the use of significantly more reading actions than the adolescents.

No gender differences were found among the children. Among the adolescents, a significant gender difference was found only in the *take* action category. The adolescent boys reported using actions in this category significantly more (45.8%) than the adolescent girls (17.1%), **χ^2^** (1) = 6.25, *p* = 0.12.

Finally, action category use was compared between the three groups of years since diagnosis. As presented in Table 3, significant differences were found in two action categories, reflecting that those who had been diagnosed for 1 to 3 years reported a significantly higher rate of *check* and *take* actions compared to the other two groups.

## 4. Discussion

Managing and adhering to a lifelong GFD, the required treatment for CD, can be challenging, especially during the complex transition from childhood to adulthood, which comes with its own unique health and developmental issues [8,9,29,30]. During this developmental process, children and adolescents with CD are gradually required to take on the responsibility from their parents for making decisions and independently self-managing their health condition [13]. Much of the literature in the CD field concerns what is safe and not safe to consume when adhering to the required GFD. Further, professional guidelines for managing CD and a growing body of literature over the past two decades address quality of life (e.g., [17,31,32]. Recently, a novel viewpoint of benchmarks was suggested. It combines the knowledge and social skills required to navigate specific tasks while managing a GFD and the sequence of acquiring learning skills in different life settings [13,33].

Health management is a daily life task that entails activities related to developing, managing, and maintaining health and wellness routines, including self-management to improve or maintain health [23]. In this study, children and adolescents with CD shared their experiences of what they do or don’t do while managing their GFD within their daily settings and environments. Examination of the data and common themes for categorization identified these daily actions. The qualitative analysis of the participants’ accounts revealed 10 categories portraying a wide range of actions these children and adolescents with CD perform in their daily interactions with their close family and social environments [12]. These 125 actions compose a list of effective strategies they use to support themselves in planning how to manage, make decisions, and conduct themselves in the many food-related activities they encounter in their daily lives despite their participation restrictions [34]. Strategies include all the methods, techniques, or procedures people use to manage their available cognitive resources to cope effectively and function in different challenging and complex situations [21]. These suggested cognitive strategies can be combined, adjusted, or modified to help others cope with cognitive challenges and to support effective functioning [21] while adhering to a GFD, self-managing, and maintaining healthy routines [23]. Exploring these self-generated do’s and don’ts provides the children and adolescents with perspectives and contexts in which they develop and prepare for life transitions [35,36].

The similarity of actions used in both age groups can reflect these children and adolescents’ ability to act on, manage, and self-advocate their needs, regardless of their age. Self-advocacy is assertiveness and willingness to represent one’s own interests in making life decisions when managing a disease or disability [23,37]. It involves knowing one’s abilities and needs to recognize problems and ways to address them, being prepared to fulfill these needs and solve problems, communicating with others to solve problems and fulfill needs, and initiating change, including negotiating with others. This study’s participants reported the highest number of actions in the first two action categories, *Tell* and *Ask*, emphasizing their vast capacity to use these actions in daily life. These actions can be specified as communication actions; indeed, self-advocacy is linked to self-knowledge, independence, and communication [38].

Previous health-related quality research encompassed children and adolescents with CD from different countries and cultures (including the current study sample). The studies found that communication (i.e., how the child feels when speaking about celiac disease) scored highest compared to other health-related quality-of-life components [39,40]. The ability to effectively function in different challenging and complex situations and sufficient self-advocacy skills are imperative for the transitional process of gaining independence from childhood through adolescence and finally to adulthood [21,37]. Furthermore, developing self-advocacy at younger ages to facilitate smoother transitions is significant [40]. Thus, the high volume in the *Tell* and *Ask* categories reflects these actions’ crucial role in the daily management of CD.

Interestingly, despite the high costs of consuming a GFD and the economic burden on those diagnosed with CD [2], the last category, *Buy* (actions related to money), included the fewest actions. Although no significant difference was found between the two age groups, *Buy* actions were used more frequently in the adolescent group than in the children group. This limited action use may convey the magnitude of the other coping actions required of these children and adolescents, regardless of the gluten-free products’ financial cost, as reflected in the 121 actions in all categories beside *Buy*. This category might expand as these adolescents transition into adulthood and have more financial responsibilities.

A comparison between the two age groups mainly revealed no significant differences. The high use of *Ask* and *Make* actions among the adolescents may reflect the strive for independence that characterizes the adolescence developmental stage and their anticipation of taking on adult roles [9,41]. Conversely, the younger group reported significantly higher use of actions in the *Read* category than the adolescents did. This finding was surprising, as it was assumed the adolescents’ reading abilities would be more advanced than the younger children’s. Nevertheless, this disparity might be explained by the fact that younger children are still mastering reading, highlighting this ability as a distinct *skill*. The adolescents might have incorporated reading into other actions, such as *Check* or *Take interest.*

Additionally, the use of actions per years since diagnosis was mostly the same. The two actions where a significant difference was found were *Check* and *Take* actions. This difference reflects that as the years since diagnosis increase, it is possible that participants’ knowledge, ability to manage their diet, and adherence to routine increase and the need to check and take food with them to food-related daily events decreases. Nevertheless, no differences were found in the use of other actions, highlighting once again that maintaining a gluten-free diet is still the only available treatment for CD, and managing and maintaining a life-long challenging strict diet continues to be necessary regardless of age or time since diagnoses.

## 5. Limitations and Future Research

This study’s results should be interpreted with caution considering the study’s limitations. First, the participants’ CD diagnoses were based on parental reports of biopsy or blood tests, and adherence to a GFD was determined via parental and self-reports. Future studies should consider collaborating with gastroenterologists and dieticians who specialize in CD to obtain medical confirmation of the diagnoses, adherence levels, and response to accidental or deliberate gluten consumption. Second, data were collected from Hebrew-speaking children and adolescents in Israel who volunteered to participate in the study. They do not necessarily represent all populations in Israel or other cultures. Third, the study included participants aged 8 to 18 yr and not young adults above the age of 18 yr. This limitation was due to the Israeli law that subjects all male and female Israeli citizens and residents to a military draft for a period of twenty-four to thirty-two months at the age of 18 [42]. The characteristics of daily routine, environment, and decision-making possibilities during military service are different to those used in a nonmilitary framework, and therefore this age group was not included in the current study. Further research could explore different cultures in different countries and include young adults. Additionally, future research can look at additional aspects of self-management, such as coping with physical, social, and psychological consequences and how to communicate best with professionals, peers, and school staff. In addition, we seemingly would expect adolescents to use strategies they have accumulated since childhood. Future research can deeply investigate strategies, characteristics, use, and change over time among children, adolescents, and adults.

## 6. Conclusions

This study’s results show the do and don’t actions that children and adolescents with CD report that they need to carry out to effectively manage their GFD in everyday life. These varied actions compose a web of cognitive planning far beyond the mere act of avoiding gluten. The do’s and don’ts generated by the children and adolescents with CD themselves can serve as an initial database of suggested strategies to support their self-management and that of others with less effective management abilities. This database can assist health professionals in building support and intervention programs to promote effective self-management and facilitate optimal adherence to the diet.

## Figures and Tables

**Table 1:** Do and don’t actions and 10 action categories.

Action Category	Action	Children (*n* = 61)	Adolescents (*n* = 66)
1. Tell	Update parents on event or activity information	13	7
	2. Tell the restaurant waiter I would like a gluten-free menu	0	1
	3. Tell the person in charge of the event that I have celiac disease	0	1
	4. Tell the person in charge that I want to see the product packaging	1	0
	5. Request that restaurants use new gloves, knives, etc.	0	1
	6. Deliver a class/school lecture to raise awareness	1	1
	7. Remind those around me not to mix or touch refreshments with gluten to avoid cross contamination	0	1
	8. Mention before dinner that I would like to serve myself first to avoid cross-contamination	0	2
	9. Remind the waiter at the restaurant not to forget that my dish is gluten-free	0	1
	10. Inform friends	0	2
	11. Say what I want to eat	3	0
	12. Draw attention to the menu when organizing an activity so that it will contain gluten-free options	1	1
	13. Say at the event that I need a gluten-free dish	0	1
	14. To be on the safe side, say I’m very sensitive when asked	0	1
	15. Share with parents when there is no gluten-free solution	0	1
	16. Remind people so not to fall between the cracks	0	2
	17. Remind those in charge to record that I have celiac disease	0	1
	18. In the case there are no gluten-free options, when eating out with friends, request that we eat somewhere else	2	0
	19. Tell others what I can and cannot eat	2	0
	20. Choose whether to share with those around me that I have celiac disease	0	1
	21. Explain to others what celiac disease is	1	2
	22. Tell person in charge to take care of gluten-free options	3	3
	23. Inform relevant parties	2	4
	24. Talk to the person in charge of the food at the event	1	0
	25. Remind those around to avoid secondary contamination	0	1
	26. Remind others that I have celiac disease and cannot eat things with gluten	2	0
	27. Tell people who do not understand what celiac is that I’m allergic	2	0
	28. Offer options for gluten-free solutions	0	1
	29. Answer and explain when people are intrigued and ask about celiac disease	0	1
	30. Remind my teacher that I exist	0	1
2. Ask	Ask questions about what is safe and unsafe	8	1
	2. Ask the person in charge/host what is gluten-free/contains gluten	3	6
	3. Ask the person I am addressing appropriately (e.g., more formally to a teacher or less formally to a friend)	0	1
	4. Ask questions in advance so as not to be disappointed	0	1
	5. Ask the person who prepared the food what the ingredients are	0	4
	6. Ask Mom/Dad/another person if you are not sure or need information	1	1
	7. Ask what dishes are gluten-free	0	3
	8. Ask someone to prepare substitutes for me before an activity with food	2	0
	9. Ask to speak to the person in charge when I am not sure about the information given to me	0	1
	10. Ask inquiry questions before activities without parents	0	3
	11. Ask who the product manufacturer is	0	5
	12. Ask the event organizer what will be served	1	3
	13. Call and ask the event organizer if there are any gluten-free accommodations	3	1
	14. Ask the person who hands out the food	1	3
	15. Ask before an activity is planned if there be refreshments or food	1	2
	16. Ask about whom I should contact about my gluten-free substitute	0	1
	17. Ask the person in charge if there is a gluten-free substitute for me	1	3
	18. Ask parents what I should take with me before an activity with food	1	1
	19. Ask another person when I cannot find information about a product	2	0
	20. Ask parents if they inquired for me before participating in an activity	0	2
	21. Ask my mother or another family member	4	1
	22. Ask my mother to check if the place has ’gluten-free options	2	1
	23. Ask before the event, “What’s in the plan?”	1	4
	24. Call the restaurant	0	3
	25. Call a friend before visiting	3	0
	26. Call friends and find out if it is suitable to go to a place with gluten-free options	0	1
	27. Call the person in charge to find out about gluten-free preparation	1	1
	28. Call to ask for gluten-free substitutes	1	0
	29. Call parents to double-check	1	0
3. Check	Check information online, on social media	2	8
	2. Check for gluten-free options at the place where an activity is scheduled	1	6
	3. Check where there are gluten-free options to arrange my activities there	1	0
	4. Make sure the information on the Internet is up-to-date and accurate	0	1
	5. Check and do not rely on someone else’s answer that the product is gluten-free	0	2
	6. Check and make sure before eating a dish that is defined as gluten-free	5	2
	7. Check by myself when eating out; rely only on myself	0	1
	8. Check in advance what is safe/not safe to be prepared accordingly	3	1
	9. Check if the menu includes gluten-free options	2	4
	10. Check in advance what will happen; don’t take any chances	0	4
	11. Check and then double-check	1	3
	12. Check in advance what needs to be brought from home	1	5
	13. Check the refreshments/food packaging	12	4
	14. Look and check what gluten-free options are available	0	1
	15. Before a vacation, check online how to say and pronounce “gluten” in the local language	0	2
	16. Check and decide in advance what to eat before eating out so as not to waste time	0	1
	17. Check and use my judgment	0	3
4. Avoid	Avoid if the ingredients say, “May contain gluten”	2	1
	2. Strictly adhere to a gluten-free diet	10	7
	3. Be sure to check, even when seeing someone else with celiac is eating something	1	1
	4. Have only a drink if there are no gluten-free options	2	1
	5. Be careful not to take any risks of eating gluten	3	4
	6. Be sure to avoid eating if I cannot check if it’s gluten-free	1	0
	7. Maintain self-discipline and do not try to eat gluten	2	1
	8. Be careful not to be tempted by foods that contain gluten	2	0
	9. Be sure to avoid eating things I am not sure do not contain gluten	1	1
	10. If unsure, do not eat	2	0
	11. Avoid eating food I am not familiar with	0	2
	12. Tell myself I came to enjoy myself and not to eat	1	1
5. Take interest	Know what is safe and what is not	2	3
	2. Keep up-to-date on celiac information	0	2
	3. Take an interest in and search for information from various sources (e.g., read online articles)	1	3
	4. Take an interest in the menu	1	2
	5. Take an interest in cooking and baking	1	0
	6. Rely on my knowledge	0	5
	7. Remember that celiac disease is a chronic health condition that does not go away, and the only treatment is strict adherence to a gluten-free diet	0	1
	8. Take responsibility	3	8
6. Take	Bring a gluten-free substitute with me	12	10
	2. Keep a box with substitutes at the place of the activity	5	0
	3. Always take a gluten-free substitute, just in case	1	2
	4. Take gluten-free bread or rolls to an activity	1	5
	5. Take basic gluten-free products for a long stay away from home	2	1
	6. Take equipment for preparing gluten-free food	1	1
	7. Take gluten-free food in a cooler bag	0	1
7. Make	Cook gluten-free food for myself	2	12
	2. Prepare reminder notes for myself	0	1
	3. Send myself cellphone reminders	0	1
	4. Prepare in advance	4	8
	5. Make sure to eat before an event with food	2	5
	6. Bake gluten-free cakes/cookies	2	6
8. Collaborate	Ask and consult with parents before an activity	0	2
	2. Coordinate bringing gluten-free food with another sensitive participant	2	1
	3. Prepare gluten-free food with someone else	6	2
	4. Talk to friends about finding places with gluten-free options	1	0
	5. Keep up-to-date with friends when planning to bring refreshments	0	1
	6. Take part in the planning of a social gathering	0	2
9. Read	Read the gluten-free options on the menu	2	0
	2. Read ingredients and allergy information on the packaging	17	5
	3. Look for the “gluten free” sign or icon	4	0
	4. When abroad, look for the words “gluten-free” or the equivalent in the local language	0	1
	5. Read information in gluten-free food guides	0	1
	6. Read ingredients and look for “contains up to 20 PPM”	1	0
10. Buy	Take a wallet with money in case you need something	0	3
	2. Join parents in shopping and choosing gluten-free products	1	0
	3. Buy gluten-free products to take with you	2	2
	4. Buy a gluten-free dish to take with you and join others at an activity	0	2

**Table 2:** Comparison of action category use between children (8–11 yr) and adolescents (12–18 yr).

Action Category	Entire Sample (*N* = 126)	Children (*n* = 61)	Adolescents (*n* = 65)	χ^2^	*p*
1. Tell	43.7%	44.3%	43.1%	0.018	0.893
2. Ask	46.8%	37.7%	55.4%	3.950	0.047
3. Check	47.6%	44.3%	50.8%	0.534	0.465
4. Avoid	30.2%	37.7%	23.1%	3.197	0.074
5. Take interest	17.5%	11.5%	23.1%	2.939	0.086
6. Take	31.7%	36.1%	27.7%	1.018	0.313
7. Make	29.4%	16.4%	41.4%	9.592	0.002
8. Collaborate	9.5%	13.1%	6.2%	1.770	0.183
9. Read	21.4%	34.4%	9.2%	11.865	0.001
10. Buy	7.1%	3.3%	10.8%	2.662	0.103

**Table 3:** Comparison of action category use between years since diagnosis.

Action Category	Entire Sample (*N* = 126)	6 Mon–1 yr (*n* = 4)	1–3 yr (*n* = 37)	>3 yr (*n* = 85)	χ^2^	*p*
1. Tell	43.7%	75.0%	45.9%	41.2%	1.889	0.389
2. Ask	46.8%	100%	43.2%	45.9%	4.763	0.092
3. Check	47.6%	0.0%	62.2%	43.5%	7.344	0.025
4. Avoid	30.2%	25.0%	43.2%	24.7%	4.258	0.119
5. Take interest	17.5%	50.0%	18.9%	15.3%	3.270	0.195
6. Take	31.7%	0.0%	45.9%	27.1%	6.165	0.046
7. Make	29.4%	50%	21.6%	31.8%	2.127	0.345
8. Collaborate	9.5%	0.0%	16.2%	7.1%	2.944	0.230
9. Read	21.4%	25.0%	18.9%	22.4%	0.212	0.899
10. Buy	7.1%	0.0%	8.1%	7.1%	0.361	0.835

## Data Availability

The data presented in this study are available on request from the author. The data are not publicly available due to ethical restrictions.
